# Validation of the 15-item Things You Do Questionnaire among South Asian adults: a psychometric analysis

**DOI:** 10.1186/s40359-026-05314-7

**Published:** 2026-08-10

**Authors:** Gary Ka-Ki Chung, Bulbul Sharma, Kai Sing Sun, Danna Camille Vargas, Hasiba Munir, Mariem Sharif, Saba Asim, Nabina Pun, Tsz Lui Tang, Man Hin Chio, Chi Yui Wong, Eliza Lai-Yi Wong, Nickolai Titov, Dong Dong, Eng-Kiong Yeoh

**Affiliations:** 1 JC School of Public Health and Primary Care, Faculty of Medicine, The Chinese University of Hong Kong, Room 419, Hong Kong, China; 2 School of Psychological Sciences, Faculty of Medicine, Health and Human Sciences, Macquarie University, Sydney, Australia

**Keywords:** Daily health behaviors, Mental health, Quality of life, Reliability, Validity, South Asian

## Abstract

**Background:**

The Things You Do Questionnaire–15 item version (TYDQ‑15) is a brief self‑report measure assessing adaptive daily behaviours associated with mental health across five behavioural domains. Although the TYDQ‑15 has demonstrated strong psychometric properties in Western populations, its validity among South Asian adults remains largely unexplored. This study aimed to evaluate the factor structure, reliability, and validity of the TYDQ‑15 among South Asian adults.

**Methods:**

A cross-sectional online survey was conducted among 765 adults from India, Pakistan, and Nepal. Participants completed the TYDQ-15 and the physical and psychological domains of the brief version of the World Health Organization Quality-of-Life Scale (WHOQOL-BREF). Internal consistency was assessed using Cronbach’s alpha, whereas construct validity was examined through confirmatory factor analysis (CFA). Convergent, discriminant, and criterion validity were evaluated using average variance extracted and composite reliability, heterotrait–monotrait ratio, and Pearson correlation with WHOQOL-BREF domains, respectively.

**Results:**

The TYDQ-15 demonstrated excellent internal consistency overall (Cronbach’s α = 0.91) and across ethnic groups. CFA supported the original five-factor structure, with acceptable to excellent model fit indices across samples (CFI = 0.94–0.98; RMSEA = 0.04–0.07). Convergent and discriminant validity were generally supported, with minor variations by ethnicity. Criterion validity was evidenced by moderate positive correlations with psychological (*r* = 0.41) and physical (*r* = 0.39) quality-of-life domains (both *p* < 0.001).

**Conclusion:**

The TYDQ-15 showed promising psychometric properties among South Asian adults from India, Pakistan, and Nepal, although further research is needed to confirm measurement invariance and cross-cultural comparability.

**Supplementary Information:**

The online version contains supplementary material available at https://doi.org/10.1186/s40359-026-05314-7.

## Introduction

A growing body of evidence suggests that engagement in specific, modifiable daily actions plays a critical role in maintaining and improving mental health [1–3]. These actions span multiple domains, including cognitive patterns, goal‑directed behaviours, lifestyle habits, and social connectedness, which individually and collectively are often targets of psychological interventions [4–6]. A recent evidence-based approach to operationalizing these behaviours in a structured and quantifiable manner has resulted in the Things You Do Questionnaire (TYDQ), a self‑reported measure designed to assess the frequency of daily actions associated with mental health [7].

The TYDQ has a theoretically coherent five‑factor structure comprising meaningful activities, healthy thinking, goals and plans, healthy routines, and social connections, which showed good reliability and validity in a large community sample of Australian adults [7]. Notably, the TYDQ score is robustly associated with mental health outcomes including depression, anxiety, and life satisfaction, supporting its relevance as a transdiagnostic behavioural indicator. It is also responsive to treatments, as change in TYDQ score was found to parallel improvements in mental health outcomes at post intervention in observational studies [8]. Recent randomized controlled trials (RCT) have demonstrated a causal link between the frequency of TYDQ actions and symptoms of depression and anxiety. One RCT demonstrated that daily nudging of the TYDQ actions resulted in symptom improvement relative to a control group in adults with elevated baseline symptoms of depression and anxiety [9]. Another RCT among asymptomatic adults found that compared to a control group, a group who restricted the TYDQ actions experienced an increase in symptoms, which resolved after they resumed the actions [10].

Nonetheless, practical constraints related to assessment burden motivated the development of shorter versions of the scale, particularly for use in digital health, population research, and repeated measurement contexts. In response, a 15‑item TYDQ (TYDQ‑15) was developed and validated using large community and clinical samples, demonstrating strong internal consistency, retention of the original five-factor structure, and superior model fit and variance explained compared to the original version [11]. Despite these strengths, existing validation studies have been conducted mostly with Australian adults, leaving the cultural generalizability of TYDQ‑15 largely unexamined. A recent exception is a study that evaluated a Turkish-language version of the TYDQ-15 with adolescent and adult participants, and which found robust psychometric properties [12].

As Titov et al. noted, further research is needed to examine the model performance in other cultural settings to ensure that the measure functions consistently across diverse populations [7]. This need is especially pertinent for the South Asian community who not only possess cultural norms and behaviour patterns that differ markedly from those typical of Western populations, but also constitute roughly a quarter of the global population and form one of the world’s largest migrant groups across developed regions [13]. Moreover, South Asians, especially the diaspora populations living in urban settings, often face unique social stressors related to migration, socioeconomic challenges, cultural adjustment, discrimination, and under-utilization of formal mental health services [14], underscoring the need for a tool to assess adaptive behaviours beyond the conventional focus on clinical mental health symptoms. As cultural context can shape patterns of daily activities and interpretation of questions, it is important to examine whether the established five-factor model fits well among South Asian communities. Thus, by validating the TYDQ-15 in South Asian adults, the present study contributes to broader efforts to support culturally appropriate, behaviour-focused mental health assessment for both South Asian communities and the wider international diaspora.

## Methods

### Study population

A survey questionnaire was designed by our team (Supplementary Material 1) and administered through Qualtrics’s online platform using its recruitment services and pre-existing pool of potential respondents. Data were collected between 9 September 2025 and 7 November 2025 across India, Pakistan, and Nepal, targeting adults aged 18 years or above. The country-wise invitation targets were 1,164 for India, 2,037 for Nepal, and 2,326 for Pakistan. Quota sampling was applied with approximately ± 5% flexibility to approximate the distribution of age and gender in respective countries. In total, 765 South Asian adults (i.e., 318 Indians, 141 Nepalese, and 306 Pakistani) were successfully recruited.

### Measurements

#### 15-item Things You Do Questionnaire

The TYDQ-15 is a self-report instrument designed to assess the frequency of daily actions linked to mental health, organized across five behavioural domains including healthy habits, healthy thinking, meaningful activities, goals and plans, and social connections. It comprises three items per domain, each rated on a 5-point Likert scale ranging from 0 (“not at all”) to 4 (“every day”). The TYDQ-15 was first translated into Hindi, Urdu, and Nepali by in-house bilingual research staff from the respective South Asian backgrounds with training in public health or related health disciplines. The translation process aimed to preserve the original meaning of each item as closely as possible. These translated versions were then reviewed and proofread by an external professional translation service to ensure linguistic accuracy and conceptual equivalence. As the original items are straightforward and do not contain culturally specific expressions or examples, no major cultural modifications were made.

#### Health-related quality of life

To assess the criterion validity, the psychological and physical domains of the brief version of the World Health Organization Quality-of-Life Scale (WHOQOL-BREF) were adopted [15]. The psychological domain measures include self-image, negative thoughts, positive attitudes, self-esteem, mentality, learning ability, memory concentration, religion, and the mental status, whereas the physical health domain includes items on mobility, daily activities, functional capacity, energy, pain, and sleep. The official English, Urdu, Nepali, and Hindi versions of the WHOQOL-BREF have been used. All items within these WHOQOL-BREF domains were rated on a 5-point ordinal scale ranging from 1 to 5, while domain-specific scores were transformed to a scaled score range of 4 to 20.

### Statistical analysis

Descriptive statistics on the basic sociodemographic characteristics of participants were reported as count data and percentages for categorical variables and mean and standard deviation (SD) for continuous variables. Also, ethnic differences were tested using analysis of variance (ANOVA) or chi-squares tests. The TYDQ-15 was validated based on the overall sample and by each South Asian group, based on reliability and validity measures as shown below:

#### Reliability

Internal consistency for the TYDQ-15 and its subscales was assessed using Cronbach’s alpha, where coefficients ≥ 0.70 were considered acceptable [16]. Corrected item–total correlations were also examined, and items with correlations < 0.30 were flagged for potential removal [17].

#### Validity

Confirmatory factor analysis (CFA) using maximum likelihood estimation with robust corrections to standard error was conducted to test the five-factor structure of the TYDQ-15. A direct confirmatory approach was adopted because the scale is an established instrument with a well-defined, a priori conceptual framework, making CFA suitable for validation studies that test whether the original model fits new data rather than exploring new dimensions [18]. Although the items were measured on ordinal Likert-type scales, a previous simulation study has shown that maximum likelihood estimation is appropriate and would perform as well as categorical least square models when the indicators comprised five or more response categories [19]. Moreover, maximum likelihood estimation facilitates the evaluation of standard model fit indices, which are commonly used to assess model adequacy in CFA.

Given the relatively large sample size (> 200), the χ² statistic and its p-value were not emphasized [20]. Model fit was evaluated using the comparative fit index (CFI), Tucker–Lewis Index (TLI), root mean square error of approximation (RMSEA), and standardized root mean square residual (SRMR). Following established guidelines, CFI and TLI values ≥ 0.95 indicated excellent fit and ≥ 0.90 indicated acceptable fit [21, 22]; RMSEA and SRMR values ≤ 0.08 were interpreted as indicative of good model fit [23]. Convergent validity was assessed using average variance extracted (AVE) and composite reliability (CR) with AVE ≥ 0.50 and CR ≥ 0.70 deemed acceptable [16]. Nonetheless, following Fornell and Larcker, constructs with AVE < 0.50 but CR ≥ 0.70 were still considered to demonstrate convergent validity [24]. Criterion validity was examined using bivariate Pearson correlations (r) of TYDQ-15 scores with WHOQOL-BREF physical and psychological domains. Discriminant validity was evaluated using the heterotrait-monotrait ratio based on geometric mean (HTMT2), which has been shown to outperform the traditional Fornell–Larcker criterion [25]. Ratios < 0.90 were interpreted as evidence of adequate discriminant validity [26]. Given the small number of missing values, listwise deletion was adopted for analyses. All statistical tests were two-tailed with a significance level of *p* < 0.05, and conducted using the statistical package Stata version 17 and R version 4.5.2.

## Results

### Descriptive statistics

Among the 765 participants, the majority were aged between 18 and 49 (85.1%) and had a sub-degree educational level or above (83.4%), with a roughly equal sex ratio (Table 1). Variations in demographic characteristics were observed across ethnic groups, with a relatively older age structure in the Pakistani sample. Also, education level tended to be lower in the Nepalese sample but higher in the Pakistani sample. The mean TYDQ-15 total score was 41.1 ± 12.0, while the mean scores of TYDQ-15 subscales ranged from 7.8 to 8.7. In addition, the mean WHOQOL-BREF psychological and physical domain scores were 14.5 ± 2.7 and 14.3 ± 2.3, respectively. Across ethnic groups, the mean scores of TYDQ-15 and its subscales, as well as the health-related quality of life, tended to be lower in the Nepalese sample compared to the other two groups.

**Table 1:** Basic demographic characteristics of the participants in the overall sample and across ethnic groups

		Total		Indian		Nepalese		Pakistani		*p*
		*N* or Mean	% or SD	*N* or Mean	% or SD	*N* or Mean	% or SD	*N* or Mean	% or SD	
Age										< 0.001
	18–29	286	37.4%	124	39.0%	76	53.9%	86	28.1%	
	30–39	166	21.7%	61	19.2%	36	25.5%	69	22.5%	
	40–49	199	26.0%	115	36.2%	26	18.4%	58	19.0%	
	50–59	73	9.5%	13	4.1%	3	2.1%	57	18.6%	
	60 or above	41	5.4%	5	1.6%	0	0.0%	36	11.8%	
Sex										0.433
	Female	399	52.2%	173	54.4%	75	53.2%	151	49.3%	
	Male	366	47.8%	145	45.6%	66	46.8%	155	50.7%	
Education										< 0.001
	Secondary or below	127	16.6%	38	11.9%	62	44.0%	27	8.8%	
	Sub-degree	225	29.4%	149	46.9%	33	23.4%	43	14.1%	
	Degree or above	413	54.0%	131	41.2%	46	32.6%	236	77.1%	
TYDQ-15 total score		*41.1*	*12.0*	*42.0*	*11.5*	*36.9*	*12.5*	*42.1*	*12.0*	< 0.001
	Meaning activities	*7.9*	*2.9*	*8.3*	*2.7*	*6.7*	*2.8*	*7.9*	*3.1*	< 0.001
	Healthy thinking	*8.5*	*2.9*	*8.2*	*2.5*	*7.6*	*3.2*	*9.1*	*2.9*	< 0.001
	Goals and plans	*8.3*	*3.0*	*8.4*	*2.6*	*8.0*	*3.5*	*8.2*	*3.3*	0.384
	Healthy routines	*8.7*	*2.9*	*8.7*	*2.6*	*8.0*	*3.4*	*9.0*	*2.9*	0.002
	Social connections	*7.8*	*3.0*	*8.3*	*2.6*	*6.7*	*3.5*	*7.8*	*3.1*	< 0.001
WHOQOL-BREF psychological domain		*14.5*	*2.7*	*14.9*	*2.4*	*13.3*	*2.7*	*14.5*	*2.8*	< 0.001
WHOQOL-BREF physical domain		*14.3*	*2.3*	*14.1*	*2.1*	*13.6*	*2.2*	*14.7*	*2.5*	< 0.001

*WHOQOL-BREF* Brief version of the World Health Organization Quality-of-Life Scale

### Reliability

In the overall sample, corrected item-total correlations of the 15 items ranged from 0.53 to 0.689, indicating that all items demonstrated adequate discrimination (Table 2). Excellent internal consistency was observed for the total scale (Cronbach’s alpha = 0.91). The five subscales also showed good internal consistency, with Cronbach’s alpha values ranging from 0.72 to 0.81. Across ethnic groups, similar level of internal consistency was observed (Cronbach’s alpha = 0.94 for Indians, 0.89 for Nepalese, and 0.90 for Pakistani). No items with a corrected item-total correlation < 0.30 were flagged for removal across samples.

**Table 2:** Corrected item-total correlations and Cronbach’s alpha if item deleted for the TYDQ-15 in the overall sample and across ethnic groups

	Overall sample		Indian		Nepalese		Pakistani	
	Corrected item- total correlations	Cronbach’s alpha if item deleted	Corrected item- total correlations	Cronbach’s alpha if item deleted	Corrected item- total correlations	Cronbach’s alpha if item deleted	Corrected item- total correlations	Cronbach’s alpha if item deleted
TYDQ_1	0.59	0.91	0.68	0.94	0.38	0.89	0.62	0.89
TYDQ_2	0.53	0.91	0.69	0.94	0.34	0.89	0.54	0.89
TYDQ_3	0.60	0.91	0.66	0.94	0.40	0.89	0.65	0.89
TYDQ_4	0.58	0.91	0.72	0.94	0.51	0.88	0.51	0.89
TYDQ_5	0.59	0.91	0.69	0.94	0.48	0.88	0.56	0.89
TYDQ_6	0.63	0.91	0.68	0.94	0.64	0.88	0.60	0.89
TYDQ_7	0.69	0.90	0.69	0.94	0.72	0.87	0.68	0.89
TYDQ_8	0.64	0.91	0.69	0.94	0.65	0.88	0.63	0.89
TYDQ_9	0.68	0.90	0.74	0.93	0.67	0.88	0.65	0.89
TYDQ_10	0.61	0.91	0.68	0.94	0.61	0.88	0.54	0.89
TYDQ_11	0.65	0.90	0.69	0.94	0.69	0.87	0.60	0.89
TYDQ_12	0.58	0.91	0.71	0.94	0.58	0.88	0.47	0.89
TYDQ_13	0.65	0.90	0.71	0.94	0.64	0.88	0.61	0.89
TYDQ_14	0.57	0.91	0.70	0.94	0.50	0.88	0.48	0.89
TYDQ_15	0.54	0.91	0.67	0.94	0.49	0.88	0.47	0.89

### CFA model fit

**Fig. 1:**
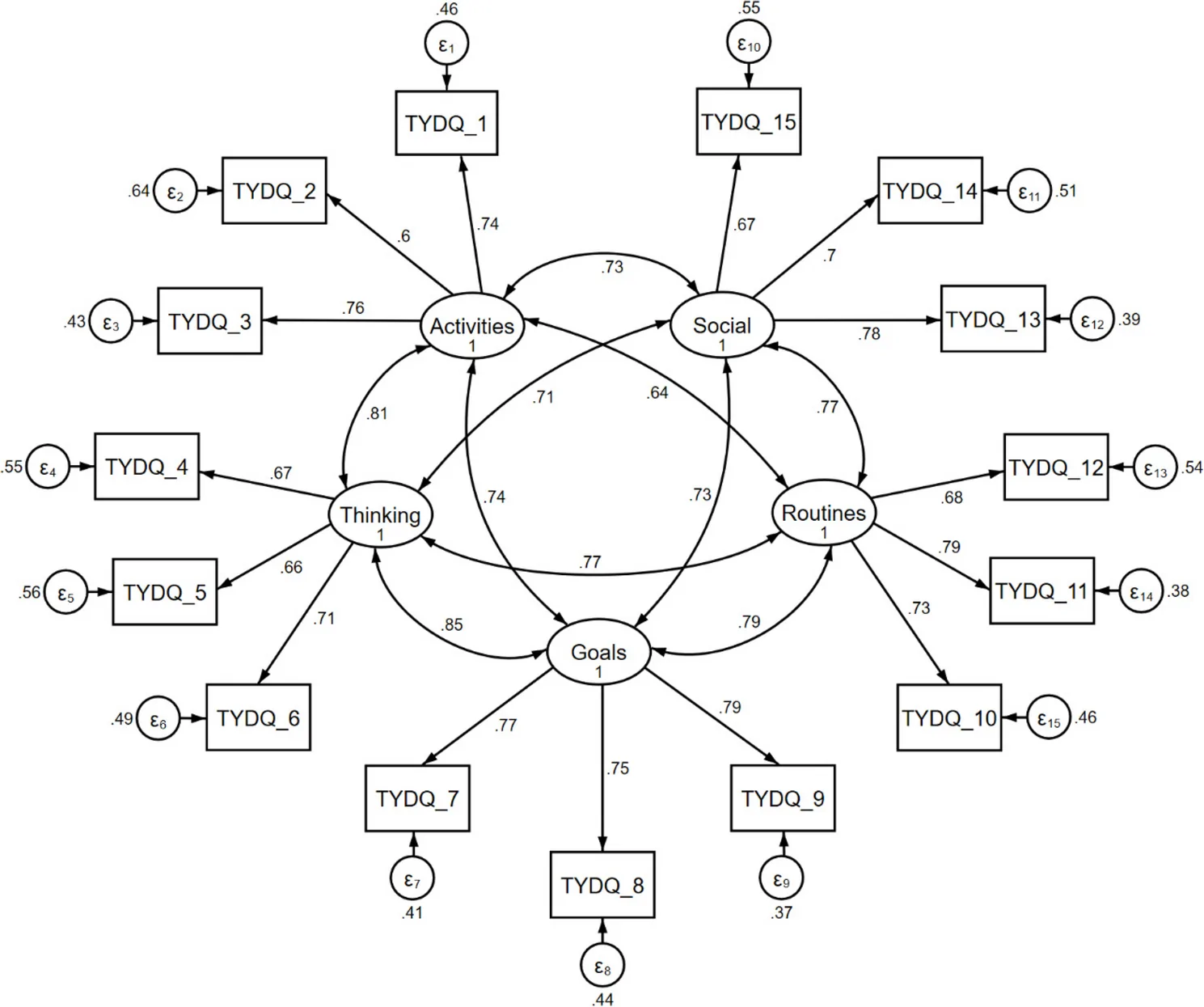
Path diagrams of the confirmatory factor analysis model for the TYDQ-15 in the overall sample

CFA based on the overall sample revealed excellent fit for the five-factor structure (Fig. 1), as reflected by CFI = 0.97, TLI = 0.96, SRMR = 0.03, and RMSEA = 0.05. Standardized factor loadings ranged from 0.60 to 0.79 while item R-squared values ranged from 0.36 to 0.63. In both measures, item 2 showed the lowest values whereas item 9 showed the highest. Across ethnic groups, the model fit of the same five-factor structure was excellent for Indians (CFI = 0.98, TLI = 0.98, SRMR = 0.03, and RMSEA = 0.04) and acceptable for both Nepalese (CFI = 0.94, TLI = 0.91, SRMR = 0.07, and RMSEA = 0.07) and Pakistani (CFI = 0.95, TLI = 0.94, SRMR = 0.04, and RMSEA = 0.06). The CFA path diagrams for each ethnic group were attached in Supplementary Material 2 (Figures S1-3).

### Convergent validity

Overall, the AVE values for the five latent constructs ranged from 0.47 to 0.59, and the CR values ranged from 0.72 to 0.81 (Table 3). Although AVE was slightly below 0.50 for the “meaningful activities” and “healthy thinking” constructs, CR for all constructs exceeded 0.70, supporting convergent validity. Similar patterns were observed across ethnic groups, except for the first two constructs in the Nepalese sample (AVE = 0.36 and CR = 0.61 for meaningful activities; AVE = 0.43 and CR = 0.69 for healthy thinking).

**Table 3:** Average-variance-extracted values (AVE) and composite reliability (CR) of TYDQ-15 constructs in the overall sample and across ethnic groups

	Overall sample		Indian		Nepalese		Pakistani	
	AVE	CR	AVE	CR	AVE	CR	AVE	CR
Meaning activities	0.49	0.74	0.58	0.80	0.36	0.61	0.51	0.76
Healthy thinking	0.47	0.72	0.50	0.75	0.43	0.69	0.45	0.71
Goals and plans	0.59	0.81	0.55	0.78	0.63	0.84	0.61	0.82
Healthy routines	0.54	0.78	0.60	0.82	0.61	0.82	0.46	0.72
Social connections	0.51	0.76	0.58	0.81	0.56	0.79	0.44	0.70

### Discriminant validity

In the overall sample, HMTM2 among constructs ranged from 0.67 to 0.87, indicating acceptable discriminant validity across subscales within the TYDQ-15 (Table 4). Across ethnic groups, HMTM2 between constructs were all below 0.90 in the Nepalese and Pakistani samples, but exceeded 0.90 in the Indian sample for a few pairs of constructs, in particular those with the “healthy thinking” construct involved (HTMT2 ranging from 0.94 to 1.05 with the other constructs) and also between the “goals and plans” and “healthy routines” constructs (HTMT2 = 0.93).

**Table 4:** Heterotrait-monotrait ratio based on geometric mean (HTMT2) between TYDQ-15 constructs in the overall sample and across ethnic groups

		Meaning activities	Healthy thinking	Goals and plans	Healthy routines	Social connections
Overall sample						
	Meaning activities	1.00				
	Healthy thinking	0.87	1.00			
	Goals and plans	0.77	0.85	1.00		
	Healthy routines	0.67	0.77	0.79	1.00	
	Social connections	0.72	0.70	0.72	0.76	1.00
Indian						
	Meaning activities	1.00				
	Healthy thinking	0.97	1.00			
	Goals and plans	0.87	1.05	1.00		
	Healthy routines	0.76	0.94	0.93	1.00	
	Social connections	0.85	0.94	0.90	0.86	1.00
Nepalese						
	Meaning activities	1.00				
	Healthy thinking	0.71	1.00			
	Goals and plans	0.63	0.85	1.00		
	Healthy routines	0.47	0.69	0.75	1.00	
	Social connections	0.36	0.51	0.60	0.72	1.00
Pakistani						
	Meaning activities	1.00				
	Healthy thinking	0.87	1.00			
	Goals and plans	0.76	0.75	1.00		
	Healthy routines	0.64	0.66	0.73	1.00	
	Social connections	0.70	0.62	0.66	0.68	1.00

### Criterion validity

Overall, bivariate correlations indicated that the TYDQ-15 total score was moderately associated with both WHOQOL-BREF domains (physical: *r* = 0.39, *p* < 0.001; psychological: *r* = 0.41, *p* < 0.001) (Table 5). Similar trends were observed for the individual subscales. Across all ethnic groups, the total score was significantly correlated with WHOQOL-BREF physical and psychological domains, despite a relatively weaker extent of correlation in the Indian sample.

**Table 5:** Bivariate correlations of TYDQ-15 total and construct scores with health-related quality of life in the overall sample and across ethnic groups

		Total score		Meaning activities		Healthy thinking		Goals and plans		Healthy routines		Social connections	
		*r*	*p*	*r*	*p*	*r*	*p*	*r*	*p*	*r*	*p*	*r*	*p*
Overall													
	WHOQOL-BREF psychological domain	0.407	< 0.001	0.347	< 0.001	0.364	< 0.001	0.303	< 0.001	0.323	< 0.001	0.324	< 0.001
	WHOQOL-BREF physical domain	0.394	< 0.001	0.313	< 0.001	0.385	< 0.001	0.275	< 0.001	0.395	< 0.001	0.245	< 0.001
Indian													
	WHOQOL-BREF psychological domain	0.190	0.001	0.137	0.015	0.172	0.002	0.152	0.006	0.211	< 0.001	0.161	0.004
	WHOQOL-BREF physical domain	0.260	< 0.001	0.123	0.028	0.265	< 0.001	0.237	< 0.001	0.305	< 0.001	0.217	< 0.001
Nepalese													
	WHOQOL-BREF psychological domain	0.576	< 0.001	0.360	< 0.001	0.540	< 0.001	0.467	< 0.001	0.379	< 0.001	0.450	< 0.001
	WHOQOL-BREF physical domain	0.410	< 0.001	0.265	0.001	0.370	< 0.001	0.217	0.010	0.454	< 0.001	0.265	0.001
Pakistani													
	WHOQOL-BREF psychological domain	0.475	< 0.001	0.447	< 0.001	0.422	< 0.001	0.328	< 0.001	0.360	< 0.001	0.322	< 0.001
	WHOQOL-BREF physical domain	0.472	< 0.001	0.456	< 0.001	0.431	< 0.001	0.333	< 0.001	0.412	< 0.001	0.241	< 0.001

*WHOQOL-BREF* Brief version of the World Health Organization Quality-of-Life Scale

## Discussion

This study provided the first evidence that the TYDQ-15 is a psychometrically sound measure for assessing adaptive daily behaviours linked to mental health among South Asian adults from India, Pakistan, and Nepal. Across the overall sample and within the three ethnic groups, the scale demonstrated good reliability, a well-supported five-factor structure, and acceptable levels of convergent, discriminant, and criterion validity. These results suggested that the core behavioural domains originally conceptualised in the TYDQ-15 remain stable and applicable in South Asian communities.

The psychometric performance observed in this study aligns with the original Australian development and validation by Bisby et al., in which the TYDQ-15 demonstrated a strong five-factor structure, excellent model fit (RMSEA = 0.04, CFI = 0.98, TFI = 0.97) and high internal consistency (Cronbach’s alpha = 0.91) in the Australian community sample [11]. The highly comparable model fit and reliability indices in our South Asian sample indicated that the measurement precision and underlying behavioural constructs of the TYDQ-15 generalize well in South Asian communities beyond the original cultural context, suggesting potential cross-cultural stability of the scale. In addition, criterion validity was indicated by moderate correlations with health-related quality of life. Similar to Bisby et al., who reported moderate associations between TYDQ-15 scores and depressive and anxiety symptoms (*r* = − 0.56 and − 0.34, respectively) [11], our findings revealed a significant correlation with psychological well-being (*r* = 0.41). Additional positive correlations with physical well-being further aligned with theoretical expectations that adaptive daily actions contribute to health and well-being [27–29].

Moreover, findings from convergent and discriminant validity analyses supported the structural integrity of the TYDQ-15. AVE and CR values were generally consistent with recommended thresholds, although the meaningful activities and healthy thinking constructs showed slightly lower AVE in the Nepalese sample. Discriminant validity was also acceptable overall, with a few exceptions in the Indian sample, particularly among constructs involving healthy thinking where HTMT2 values exceeded recommended limits. These patterns suggest that while the underlying factor model is sound, some domains may overlap conceptually in specific cultural groups, potentially reflecting cultural or linguistic variations in how certain adaptive behaviours are understood [30]. Notably, constructs involving reflective or cognitive processes, in particular healthy thinking and appraisal of meaning, may differ in salience, interpretation, or linguistic equivalence across ethnic groups [31]. These observations underscore the importance of culturally informed adaptation procedures and point to possible areas where further refinement may improve scale functioning in other linguistic and cultural contexts.

Apart from its psychometric robustness, the TYDQ-15 has important public health implications in the South Asian community given the substantial mental health burden, since the behaviour-based indicators captured in the TYDQ-15 offer a practical means of identifying early changes in daily functioning that are known to precede declines in mental well-being. Specifically, the brevity of the scale enables frontline health workers to administer it efficiently in time-limited community settings, while its non-stigmatizing focus on everyday behaviours, rather than clinical symptoms, not only enhances acceptability but also reduces the likelihood of social desirability bias. Hence, by providing a reliable and culturally acceptable indicator of adaptive daily practices, the TYDQ-15 could serve as a useful tool for community-based mental health screening and for evaluating preventive or lifestyle-based interventions in South Asian communities.

Notwithstanding the generally positive validation findings, several limitations of this study should be acknowledged. First, this study relied on a non-probability sampling approach using an online convenience sample, which may introduce selection bias particularly due to potential digital access barriers among individuals with lower socioeconomic status or older age. As the sample was restricted to three major ethnicities and relatively young, generalisability of our findings may be lower for older populations and the broader South Asian community. Second, although subgroup analyses were conducted, the sample sizes were not equal across ethnic groups, making invariance testing difficult. As configural and scalar models did not converge in attempts for comparison by ethnicity using multi-group CFA, we reported separate estimates on factor loading and model fit indices across the three ethnic groups. Third, while the psychological domain of the WHOQOL-BREF was used, this study did not include specific mental health measures, such as depression and anxiety, for a more robust evaluation of criterion validity of the TYDQ-15. Fourth, as test–retest reliability was not assessed, the temporal stability of the TYDQ-15 remains unknown. Fifth, the use of self-report measurements may be subject to recall and social desirability biases, which may influence participants’ responses and potentially distort the associations among constructs or between constructs and health-related quality of life. Last, due to the cross-sectional nature of this validation study, temporal sequence and causal inference between the TYDQ-15 and health-related quality of life could not be established. Longitudinal studies are warranted to examine its predictive validity, particularly for mental health outcomes or intervention responsiveness.

To conclude, among South Asian adults from India, Pakistan, and Nepal, the multi-dimensional TYDQ-15 scale demonstrated good reliability and model fit of the five-factor structure, in addition to reasonable levels of convergent, discriminant, and criterion validity despite slight variations across ethnic groups. However, further research is needed to confirm measurement invariance and comparability across ethnic groups. Given its potential cross-cultural applicability, additional adaptation and validation studies are warranted to assess the psychometric properties of the TYDQ-15 in other languages or settings.

## Supplementary Information

Supplementary Material 1.

## Data Availability

The datasets used and/or analysed during the current study are available from the corresponding author on reasonable request.
