# Supplementary material for: Validation of the 15-item Things You Do Questionnaire among South Asian adults: a psychometric analysis
Source: BMC Psychol. 2026 Aug 10;14:1600. doi: 10.1186/s40359-026-05314-7 (PMC13737536; doi:10.1186/s40359-026-05314-7)
Supplement: Supplementary file 1 [file 40359_2026_5314_MOESM1_ESM.docx]

**Figure S1. Path diagrams of the confirmatory factor analyses model for the TYDQ-15 in the Indian sample**

**Figure S2. Path diagrams of the confirmatory factor analyses model for the TYDQ-15 in the Nepalese sample**

**Figure S3. Path diagrams of the confirmatory factor analyses model for the TYDQ-15 in the Pakistani sample**
